# Palliative repeated electroporations of oral tumours in dogs: A case series

**DOI:** 10.3389/fvets.2022.1004811

**Published:** 2022-11-10

**Authors:** Giulia Moretti, Alfredo Dentini, Francesca Beccati, Rolando Arcelli, Irene Di Matteo, Giuseppe Giovannini, Antonello Bufalari

**Affiliations:** ^1^Department of Veterinary Medicine, University of Perugia, Perugia, Italy; ^2^Tyrus Veterinary Clinic, Terni, Italy

**Keywords:** electrochemotherapy, oral cavity tumour, dog, repeated electroporations, neoplasia, oncology

## Abstract

Electrochemotherapy (ECT) is a highly developed treatment for many solid tumours that provides good local control in 80% of neoplasms in dogs. ECT can be used to treat different types of tumours, particularly as an innovative approach for non-resectable masses. As reported in the literature, electroporation-based treatments are safe, simple, fast and cost-effective treatment alternatives for selected oral and maxillofacial tumours not involving the bone in dogs (e.g., small squamous cell carcinoma or malignant melanoma). In this descriptive retrospective paper, the authors describe the outcome of various types of oral tumours treated with ECT as a palliative first line treatment or as a rescue treatment in dogs with local tumour recurrence. Nineteen dogs were included and treated with at least one session of three electroporations coupled with intravenous administration of bleomycin every 21 days. Tumour size, localization, histotype, stage, recurrence, solid tumour response evaluation criteria (RECIST), local toxicity, progression free survival (PFS) and median survival time (MST) were evaluated. The small population did not allow the analysis of the ECT response by comparing different tumour types; further studies with a larger caseload are needed. However, all dogs, despite the low MST, showed a good local response to treatment with a rapid improvement in quality of life from the first ECT application; no side effects attributable to chemotherapy have been detected and toxicity due to the electroporation was minimal and well tolerated in all dogs.

## Introduction

Oral tumours are common in dogs, accounting for up to 6% of all tumours in this species (1–5). In dogs, the most common malignant oral tumours are, in descending order, malignant melanoma (MM), squamous cell carcinoma (SCC), and fibrosarcoma (FSA) (5, 6). Clinical signs vary from mild/moderate (increased salivation, exophthalmos or facial swelling, epistaxis, and halitosis) to more severe (bloody oral discharge, weight loss, dysphagia or pain on opening the mouth, or occasionally cervical lymphadenopathy).

Loose teeth, especially in an animal with generally good dentition, should alert the clinician to possible underlying neoplastic bone lysis (5). Surgery and radiotherapy (RT) are the two most common treatments applied for the local control of oral cancers. Aggressive surgeries (mandibulectomy, maxillectomy or orbitectomy) are generally well tolerated by dogs (5, 6). However, RT can be effective for locoregional control of oral cancers as a primary treatment, as palliative or curative treatment or as adjunctive therapy for incompletely resected masses, or as an adjunct for locally aggressive tumours (such as MM, SCC or FSA), regardless of the completeness of excision. Chemotherapy may be indicated for some tumours with higher metastatic potential (MM, osteosarcoma, FSA), with the aim of containing the metastatic spread (5). Other local ablative treatment options have been investigated, and electrochemotherapy (ECT) seems to have encouraging results (7–10). The use of ECT has been widely studied in human medicine, especially for local control of oral cavity tumours and for neoplasms of the head and neck region (11–13). Recently, ECT has also been investigated as a treatment for oral cavity tumours in dogs, in particular for MM and SCC (8, 9). The use of ECT combined with administration of chemotherapy drugs (cisplatin or bleomycin, or a combination of the two), has also been tested for several tumour types (14–17).

The purpose of this case series is to describe a population of dogs with several types of oral cavity cancer treated with repeated application of ECT coupled with intravenous bleomycin administration as a palliative treatment. The authors' aim was to evaluate retrospectively any possible differences among different tumour types in terms of treatment tolerability, local response, survival time, and local recurrence following the ECT treatment.

## Materials and methods

A cross-sectional observational study was carried out according to the STROBE checklist (18). In this retrospective case series, dogs admitted to Tyrus Veterinary Clinic of Terni with various types of oral tumour and treated with ECT between January 2016 and July 2021 were included. Dogs with at least 6 months of clinical follow-up available were included. No restriction was imposed with regard to breed, gender, age, or neutering. Patients with incomplete data were excluded.

The inclusion criteria included the availability of a complete staging (5, 19) of the tumour obtained by cytology and incisional biopsy of the mass, a complete hamatological profile (complete blood count, biochemical profile) evaluation of head and neck lymph nodes (external examination, cytology and CT evaluation) and diagnostic imaging (three-view thoracic radiographs and/or total body computed tomography). The following data were reviewed from the clinical records of the dogs: breed, sex, age, neutering, tumour grading, tumour type, clinical staging, tumour site (classified as “caudal” for tumours located in the caudal third of the hard palate, soft palate, oropharynx, angle of the mandible, tonsillar region, as “maxilla” for tumours involved the maxilla region, as “mandible” for tumours involved the mandible, as “oral mucosa” for tumours involved the mucosal part of the cheeks or lips and “tongue” for tumours involving the tongue), tumour dimension (measured manually with callipers) and treatment protocol. Local response was evaluated following RECIST criteria for solid tumours obtained after the ECT treatment protocol at 1month follow up: complete remission (CR) as total resolution of the tumour, partial remission (PR) as ≥30% reduction in tumour diameter, stable disease (SD) <30% reduction of tumour diameter or <20% increase of tumour diameter, progressive disease (PD) ≥20% increase in tumour diameter (20, 21). Toxicity due to electroporation was evaluated at the first clinical follow-up, using the grading system described by Lowe et al. (14). All follow-ups were made retrospectively, based on the recorded data and when possible with a clinical examination of the dog or at least by phone contact with the owners. All the data collected were reported as descriptive. The median survival time (MST) was calculated from the first ECT treatment to the death of the patient or to the end of the study (July 2021). Progression free survival (PFS) was calculated from the time of the first ECT treatment to the first recurrence or to the death of the animal, or alternatively we right censored the length of the PFS to the date of the end of the study.

### Statistical analysis

Data were analysed with the appropriate software (JASP Team^1^ and R Core Team^2^) Descriptive statistics for the following parameters were obtained: breed, sex, age, spaying status, tumour at first presentation or tumour recurrence, tumour localization, clinical staging, tumour size, bone involvement, number of ECT sessions, post ECT recurrence, cause of death, RECIST criteria, tumour histotype, toxicity grading, MST, DFI and tumour size reduction rate. The Shapiro-Wilk test was used to verify normality, the Levene test was used to verify equality of variances for continual variables, and statistical tests were applied as appropriate. Difference between primary tumours (group P) or recurrences (group R) treated with ECT, were evaluated by chi-square test for qualitative variables (breed, sex, spaying status, tumour localization, clinical staging, tumour size, bone involvement, number of ECT sessions, post ECT recurrence, cause of death, RECIST criteria, tumour histotype and toxicity grading) and Student-*t*-test or Mann-Whitney test as appropriate for quantitative variables (age, MST, DFI and size reduction rate). The Kaplan-Meier method was used for the time to event analysis, including DFI and MST. The log-rank test was used for differences between group F and group R in MST and DFI. The significance threshold was *p* < 0.05.

## Results

Nineteen dogs were included retrospectively: nine (48%) cross-breeds, two (11%) Golden Retrievers, two (11%) English Setters and one (5%) dog representative of each of the following breeds: Epagneul Breton, Cocker Spaniel, Cavalier King Charles Spaniel, Labrador Retriever, Pitbull and Pointer. Twelve (63%) dogs were female, and the remaining seven (37%) were male; eleven (58%) dogs were neutered, while eight (42%) dogs were not. The median age was 13 years (range: 8–17 years), median observation time was 240 days (range: 60–365), and median follow-up time was 180 days (range: 180–365). Seven (37%) patients presented a local recurrence from a previous treatment (surgery) by the referring veterinarian and the remaining 12 (63%) cases were tumours at first presentation.

Table 1 shows patient data for tumour histotype, tumour size, electrical parameters, ECT toxicity grade, adjunctive ECT session, RECIST criteria and remission rate (%).

**Table 1:** Patient data for tumour histotype, tumour size, TNM staging system, tumour bone involvement (BI), tumour recurrences treated (TR), electrical parameters (EP), ECT toxicity grade (Tox), adjunctive ECT session (Adj ECT), total number of ECT session, RECIST evaluation criteria obtained after the ECT treatment protocol at 1 month follow up, tumour size reduction rate (%), tumour recurrence after electroporation and median survival time (MST).

**n**	**Tumour histotype**	**Size**	**TNM**	**BI**	**TR**	**EP**	**Tox**	**Adj ECT**	**ECT session**	**RECIST**	**Size reduction (%)**	**Post ECT recurrence (PFS days)**	**MST (days)**
1	Melanoma	L	T3N1bM0	N	N	HE	2	Y	4	CR	100	Y (180)	365
2	Melanoma	L	T3N1bM1	N	N	HE	2	Y	4	PR	90	N	270
3	Melanoma	L	T3N1bM0	N	Y	LI	2	N	3	CR	100	N	300
4	Melanoma	L	T3N1bM0	N	Y	LI	2	N	3	CR	100	N	150
5	Melanoma	L	T3N1bM0	N	N	LI	2	N	3	CR	100	Y (300)	300
6	Melanoma	L	T3N1bM1	N	N	LI	2	N	3	PR	90	N	120
7	Melanoma	L	T3N1bM0	Y	Y	HE	2	N	3	PR	50	N	210
8	Melanoma	L	T2N1bM0	Y	Y	HE	2	N	3	PR	90	N	150
9	Melanoma	L	T3N1bM0	N	Y	HE	2	N	3	PR	90	N	300
10	Melanoma	L	T3N1bM0	Y	N	HE	2	N	3	PR	80	N	270
11	Melanoma	L	T3N0M0	N	Y	LI	2	Y	4	CR	100	Y (120)	180
12	Melanoma	L	T3N0M0	N	N	HE	5	N	3	CR	100	Y (120)	120
13	Melanoma	L	T3N1bM0	N	Y	HE	2	N	3	CR	100	N	120
14	SCC	S	T2N1bM0	N	N	HE	2	N	3	PR	90	Y (240)	240
15	SCC	L	T3N3M0	N	N	LI	2	N	3	PR	60	N	210
16	A Sarcoma	L	T3N0M0	N	N	HE	1	N	3	PR	50	N	365
17	A Sarcoma	L	T3N0M0	N	N	HE	2	N	3	CR	100	Y (60)	120
18	Fibrosarcoma	L	T3N0M0	N	N	LI	2	Y	4	PR	50	N	330
19	T Lymphoma	S	WHO I extranodal	N	N	HE	2	N	3	CR	100	Y (300)	300

BI, bone involvement; *TR*, tumour recurrence; EP, electrical parameter; Tox, Toxicity; Adj ECT, adjunctive session of ECT after the first line protocol; MST, median survival time; SCC, squamous cell carcinoma; A Sarcoma, anaplastic sarcoma; L, large tumour, >5 cm; S, small tumour < 5 cm; HE, hexagonal probe: 4 pulse, frequency 5,000 Hz, amplitude 730 V, length 100 μs; LI, linear probe: 8 pulse, frequency 5,000 Hz, amplitude 400 V, length 100 μs; Y, yes; N, no; CR, complete remission; PR, partial remission.

The tumour histotypes were: malignant melanoma (13; 68%), squamous cell carcinoma (2; 11%), anaplastic sarcoma (2; 11%), oral fibrosarcoma (1; 5%) and T-cell lymphoma (1; 5%). Complete staging was achieved in all patients: fifteen dogs were stage III (79%), while two (11%) were stage IV and one (5%) each for stage I and II, respectively; details of the TNM staging system were summarized in Table 1. Seventeen tumours (89%) were large (i.e., ≥5 cm on the largest diameter) while the remaining two (11%) were smaller than 5 cm. Seven tumours (37%) were located in the caudal part of the oral cavity (caudal third of the hard palate, soft palate, oropharynx, angle of the mandible, tonsillar region), four (21%) were located in the maxilla, three (16%) in the mandible, four (21%) in the oral mucosa (the mucosal part of the cheeks and lips) and the remaining one (5%) on the tongue; among all cases only three dogs (16%), all affected by oral melanoma, had bone involvement.

All dogs received the same ECT protocol which consisted of at least three sessions of electroporation with bleomycin as monotherapy. Bleomycin was administered intravenously at a dose of 20 mg/m^2^ of body surface area (15), and electroporation was applied 8 min after the drug administration. Three total treatment sessions every 3 weeks were applied as a first line protocol, one additional ECT session was performed only in four cases (21%) to achieve more local control of the mass. Electroporation was performed using a Cliniporator^®^ (IGEA S.p.a) by means of two types of needle electrodes with different settings for the electrical parameters mainly depending on the size and localization of the tumour: a linear probe (electrical parameters: 8 train of pulse, frequency 5,000 Hz, amplitude 400 V, length 100 μs) was used for seven (37%) tumours which were smaller than 5 cm and for those situated in the caudal part of the oral cavity (from the caudal third of the hard palate, soft palate, oropharynx, angle of the mandible, tonsillar region); a hexagonal probe (electrical parameters: 4 train of pulse, frequency 5,000 Hz, amplitude 730 V, length 100 μs) was used for twelve tumours (63%) which were located aborally or were >5 cm in the largest diameter. From all the clinical records analysed, all the ECT treatments were performed under general anaesthesia with the same anaesthetic protocol: dogs were premedicated with intramuscular butorphanol at 0.2 mg/kg, and general anaesthesia was induced with intravenous (IV) propofol 2–4 mg/kg. Maintenance after orotracheal intubation was performed by a mixture of isoflurane in 100% oxygen. A bolus of IV fentanyl (4 mcg/kg) was administered 4 min before application of the electric impulse. After the procedure, dogs received a standard analgesic and NSAID protocol (tramadol 2 mg/kg and meloxicam 0.1 mg/kg once per day) for 5 days.

**Figure 1:**
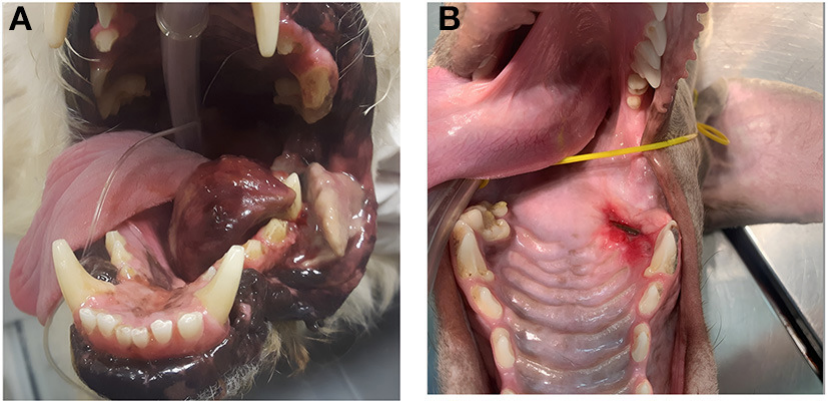
Toxicity related effects post ECT treatment: **(A)** case *n* 12, a dog with a large oral malignant melanoma after the first electroporation during the first follow-up, showing severe tumour necrosis on the lateral aspect of the left mandible (grade 5 toxicity); **(B)** case *n* 17 showing a grade 2 toxicity and local stomatitis.

**Figure 2:**
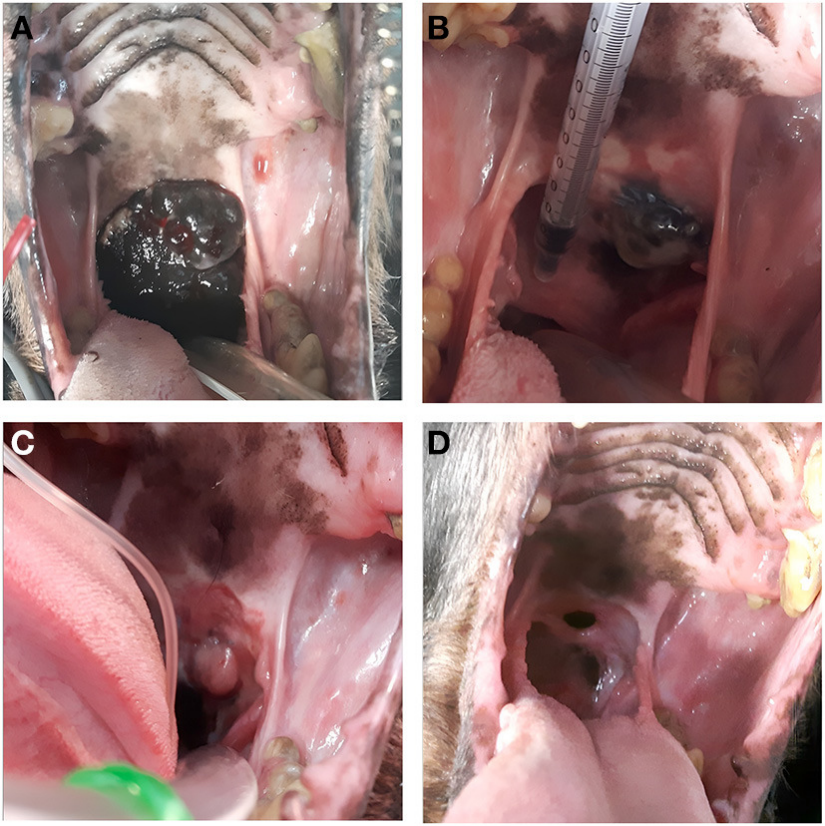
Case *n* 5, a dog with oral malignant melanoma before the ECT treatment **(A)**, after the first electroporation (21 days follow-up) **(B)**, after the second electroporation (42 days follow-up) **(C)** and at the end of the treatment (1 month follow-up) showing an oronasal fistula **(D)**.

**Figure 3:**
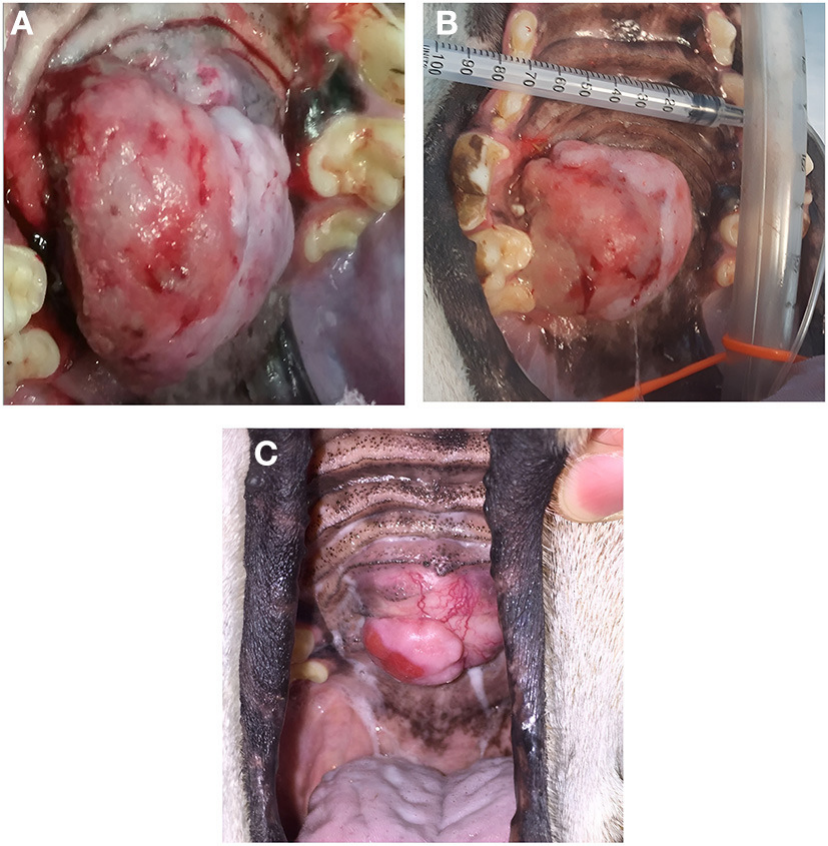
Case *n* 18, a dog with an oral fibrosarcoma, before the ECT treatment **(A)**, after the three sessions of ECT treatment **(B)**; the patient underwent also an additional ECT session showing a PR at 2 month follow-up but with a good quality of life **(C)**.

In dogs that showed toxicity-related events (swelling, stomatitis, necrosis, oronasal fistula) a symptomatic treatment was performed with cleaning and lavage of the treated area with sterile saline solution if any necrotic debrides were noted and also with local application of chlorhexidine gel 0.12% (ICF Stomodine gel) BID until healing (Figure 1). ECT toxicity of the oral mucosa was noted mainly at the first clinical follow-up after the first ECT session (Table 2) without any signs of pain or dysphagia for the dogs treated and resolved completely in almost a week. Two (11%) dogs showed transient oronasal fistula resolved spontaneously with tissue regrowth: the two dogs underwent a course of antibiotic therapy to treat a mild aspiration pneumonia due to the fistula (Figure 2). Seventeen (90%) dogs showed grade 2 toxicity, one (5%) dog showed grade 1 and another (5%) had grade 5 toxicity (13). From the recorded data, follow-ups were made at varying intervals: dogs were checked depending on the severity of the wound and the necrosis expected, with an average 4-day interval for the first few weeks and from the second ECT session every 3 weeks until complete healing: other follow-ups were performed at 1, 3, and 6 months after the first ECT treatment, and for two dogs (10%) it was possible to perform also 1 year follow-up (Table 2). Median follow-up time was 180 days, with thirteen (68%) of the dogs reaching the 6 months follow-up alive. The MST and PFS for the study population were 240 (range: 120–365 days) and 210 days (range: 30–330 days), respectively. Two (11%) dogs were still alive at the time of writing of this manuscript, while the remaining seventeen (89%) were dead; of these eleven (65%) dogs had a tumour-related death and the remaining six (35%) died from other causes. With regard to the RECIST criteria parameters, ten (53%) dogs showed PR and the remaining nine (47%) show a CR (17); the response rate after ECT based on tumour size was 100% for nine (47%) dogs, 90% for five (26%) dogs, 80% for one (5%) dog, 60% for one (5%) dog and 50% for three (17%) dogs (Figure 3). Patient data including clinical stage, tumour localization, MST, DFI, PFS and outcome are summarized in Table 3, and stratified for tumour histotype.

**Table 2:** scheduling of follow-ups and ECT sessions.

**Days**	**0**	**3–4**	**21**	**42**	**60**	**120**	**180**	**365**
Session	1°ECT	–	2°ECT	3°ECT	4° ECT (optional)	–	–	–
Follow-up	X	X	X	X	X	X	X	X (optional)

**Table 3:** Patients' data for clinical stage, tumour localization, MST, DFI, outcome and tumour-related death grouped for tumour histotype.

	**Melanoma (*n* = 13)**	**SCC (*n* = 2)**	**A Sarcoma (*n* = 2)**	**Fibrosarcoma (*n* = 1)**	**T Lymphoma (*n* = 1)**
**Clinical stage (** ***n*** **; %)**					
I	–	–	–	–	1 (100%)
II	2 (15%)	–	–	–	–
III	9 (70%)	2 (100%)	2 (100%)	1 (100%)	–
IV	2 (15%)	–	–	–	–
**Localization (** ***n*** **; %)**					
Caudal	5 (39%)	1 (50%)	–	1 (100%)	–
Maxilla	3 (23%)	–	–	–	1 (100%)
Mandibula	3 (23%)	–	–	–	–
Oral mucosa	2 (15%)	–	2 (100%)	–	–
Tongue	–	1 (50%)	–	–	–
MST days (median; range)	210 (120–365)	225 (210–240)	243 (120–365)	330	300
PFS days (median; range)	180 (120–300)	225 (210–240)	75 (60–90)	330	300
**Outcome (** ***n*** **; %)**					
Alive	1 (8%)	–	1 (50%)	–	–
Death	12 (92%)	2 (100%)	1 (50%)	1 (100%)	1 (100%)
Tumour-related death (*n*; %)	10 (77%)	–	1 (50%)	–	–

SCC, squamous cell carcinoma; A sarcoma, Anaplastic sarcoma; MST, median survival time; PFS, progression free survival.

**Figure 4:**
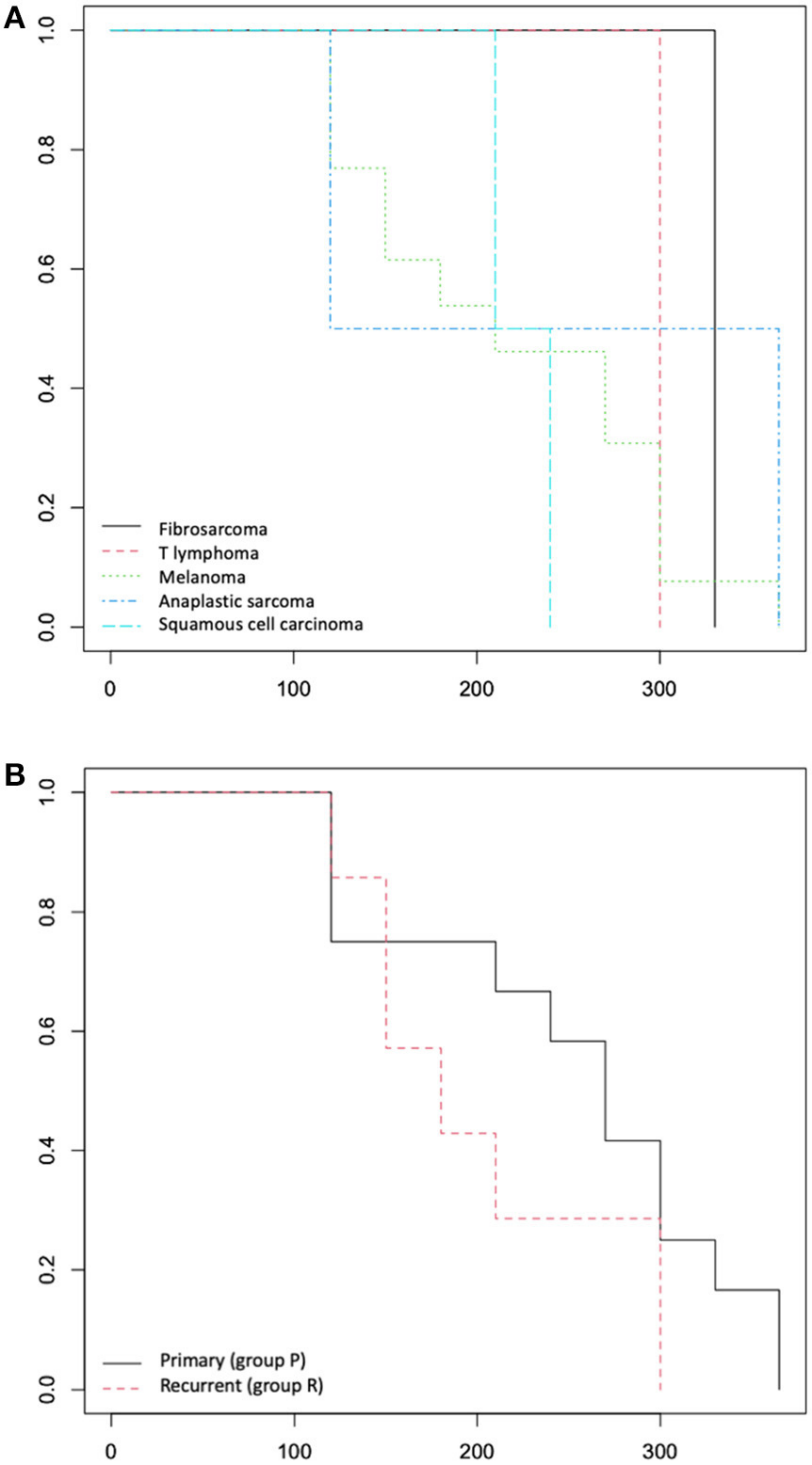
**(A)** Overall survival time: Kaplan-Meier curve according to tumour histotype and **(B)** according to primary (group P) or recurrence (group R) lesions.

Significant differences from the chi-square analysis between group P and group R were observed only for the presence of tumour bone involvement (*p* = 0.013); no significant differences were recorded in the other variables. The survival analysis didn't show any difference between the group P and group R for MST and DFI (Figure 4).

## Discussion

Electroporation-based treatments have been proven to be safe and effective in veterinary oncology, although they have not been accepted as standard treatments, especially in oral and maxillofacial oncology (22). As previously reported in the literature, ECT is a treatment recommended mostly in cases in which the owners decline surgery and/or radiotherapy or in case of standard treatments failure (23). In agreement with that finding, in this study the choice to use ECT with a palliative intent, instead of other treatments, was based on the owners' consent: the owners of all the dogs included declined first line protocols such as surgery with wide margins and/or radiation therapy, especially if the tumour was considered unresectable. The aim of this study was to describe retrospectively a heterogeneous population of oral tumours treated palliative with ECT and evaluate any differences between the different tumours histotype, in terms of treatment tolerability, local response, survival time, and local recurrence rate, in order to collect more specific indications regarding the proper use and the efficacy of ECT treatment for different oral cavity tumours; unfortunately, the low number of cases reported did not allow a proper comparison of ECT effectiveness for different tumour types. Most of the tumours included (16; 68%) were oral MM: their MST compared with a previous study (24) showed a similar value (210 and 180 days, respectively). In particular, the study of Tellado et al. (9) reported a MST of 7.5 and 4.5 months for stage III and IV, respectively, and a median time to progression of 4 months for stage III and IV oral MM; these results are in accordance with those reported in this clinical study for ECT-treated oral MM. Moreover, slightly better results are reported for the overall local response rate (ORR), which was 100%, with seven CR and six PR, compared with those of Tellado et al. (9), which showed an ORR of 70.1%, with 20.9% CR and 43.9% PR, 16.4% PD and 13.4% SD. The other tumour types were not very representative and showed different results, which should be interpreted with caution because the low number of cases. A previous study evaluated the efficacy of ECT treatment in canine oral non-tonsillar SCC (8), reporting a response rate of 90.9% with a recurrence rate of 27.3%. Dissimilar results were obtained in our study; the two cases of oral SCC showed both PR and response rate of 75%. In this study, the ECT toxicity recorded was quite low (grade 2); only one case, a dog with a large oral MM, reported grade 5 toxicity, which indicates severe swelling and tissue loss; the effects of the toxicity resolved without any type of adjunctive surgical approach, just with local daily medication for few days. This high level of toxicity was probably due to the “vascular lock” effect causing enhanced ischaemia and necrosis, also we could speculate that more necrosis occurs in larger tumours because of a larger volume of dying tumour cells, even if Lowe et al. (14) reported no relationship between tumour size and grade of toxicity. The “vascular lock” is a positive factor of ECT because the transient anti-vascular effect on the tumour allows a decrease in vascular flow within the tumour, with consequent retention of the chemotherapy drug, and also controls bleeding in haemorrhagic tumours such as mucosal cancers. Obviously, ECT toxicity cannot be evaluated in tumours treated surgically, but it may be estimated from post-surgical complications. Sarowitz et al. (25) reported that the most frequent post-surgery complications of oral tumours included wound dehiscence, oronasal fistula, pin migration, nasal discharge or aspiration pneumonia. In our study, in two cases with the tumour located in the caudal part of the oral cavity, especially on the palate, it was possible to note a transient oronasal fistula after the last ECT application; in both cases the fistula resolved without other local treatment with a regrowth of granulation tissue in almost 2 weeks. For tumours treated with ECT, most of the reports in the literature suggest that smaller diameter, a rostral localization and tumours not involving the bone are favorable prognostic factors (5, 9, 22). This is probably because tumours in rostral locations are usually detected at an earlier stage and are more likely to be resectable with complete margins or treated properly with ECT. In our study the tumour dimension was a parameter that was not evaluable because most (85%) of the dogs had a large tumour (>5 cm in the largest diameter), with only two cases (15%) in which the tumour size was smaller.

Regarding the tumour recurrences treated (12% of the cases included), it is known that tumour recurrences have a different outcomes than tumours in the first presentation, affecting negatively the outcome as they are often more aggressive and difficult to treat (2, 5, 22). The survival analysis didn't show significance between tumours treated with ECT as a first time presentation (group P) or as tumour recurrences (group R) even if the median value of MST and DFI are lower in group R (180 and 150 days respectively) than in group P (270 and 220 days respectively). The authors highlighted a potential bias of the result obtained from the survival analysis due to the heterogeneity of the tumour histotypes included in the two groups; the different tumour type may could influence the MST and DFI with a greater weight than being a primary lesion or a recurrence. Nowadays, ECT is rarely used as a first line protocol and only in case of rescue therapy, probably because there is still more to investigate about its effectiveness. One future prospective could be the evaluation of the ECT treatment for the management of local recurrences since it gives excellent results in a such difficult area as the oral cavity even when other local therapies have poor effectiveness. Further studies with a larger population are needed to prove this thesis.

In this case series only three dogs (16%) affected by oral melanoma, had bone involvement and two of them were tumour recurrences before starting the ECT protocol: they all had a PR but without any tumour progression after the ECT treatment. Bone involvement is a negative prognostic factor for oral tumours treated with ECT (8, 9, 22): in one case of this study we also observed that the tumour recurrence after ECT treatment started from the bone and not from the soft tissue of the oral cavity, as if to underline that one of the limit of the ECT is in fact reaching the deeper tissues. Moreover from the statistical analysis significant difference between primary and recurrent lesions was recorded only for tumour bone involvement (*p* = 0.013), as to support the hypothesis that tumour recurrence and bone involvement are two factors linked to each other that negatively influenced the outcome of ECT treated oral tumours. Unfortunately this results should be supported by more recruited clinical cases categorized by tumour histotype to have scientific relevance.

Despite the heterogeneity of the tumour types included this study, the results confirm that electroporation-based treatments are safe, simple, fast and effective treatment alternatives for selected oral tumours, but there is currently no consensus on timing and the quantity of retreatments (22); further studies are needed to standardize ECT protocols.

One of the limitations of this study is related to the retrospective design and the small population included, resulting in the inability to perform any statistical analysis. Moreover, the lack of randomization of treatment group and the criteria for assigning the ECT treatment instead of standard radical treatment (surgery and/or radiotherapy) led necessarily to a selection bias, including a population of tumours with negative prognostic factors (e.g., high clinical stage, large size tumours, non-resectability of masses).

Moreover the authors highlighted a potential bias regarding the toxicity grading system by Lowe et al. (14), that was developed for tumours affecting the skin: it was therefore been applied in this study to mucosal tumour of the oral cavity, that probably respond differently to the ECT treatment, even if from the results obtained from this study the toxicity recorded was generally low, similar to other studies (14, 16).

In conclusion, ECT treatment should be considered as an alternative treatment for non-resectable oral cavity tumours, especially when owners have concerns about the financial burden and aesthetic outcome that usually follows radiation therapy or surgery. Prospective case-control studies are required to better understand the effectiveness of ECT for oral tumours, with randomized treatment groups and comparing electroporation with surgery or irradiation or both, also analysing the influence of various prognostic factors in treatment response. It would be of interest to evaluate the role of intraoperative ECT (electroporation post-surgical debulking) in oral and maxillofacial tumours, which might allow a more conservative surgery, especially for unresectable tumours, leading to faster relief of clinical signs and at the same time reducing tumour progression.

## Data availability statement

The raw data supporting the conclusions of this article will be made available by the authors, without undue reservation.

## Ethics statement

Ethical review and approval was not required for the animal study because it is a retrospective case series. Written informed consent was obtained from the owners for the participation of their animals in this study.

## Author contributions

AD, GG, and IM performed the electroporation treatment of all the cases included. GM, FB, AB, AD, and RA performed the research design, review, and structuring of the article. GM, IM, and GG performed data collection and tabulation. FB performed the statistical analysis. All authors contributed to the article and approved the submitted version.

## Conflict of interest

The authors declare that the research was conducted in the absence of any commercial or financial relationships that could be construed as a potential conflict of interest.

## Publisher's note

All claims expressed in this article are solely those of the authors and do not necessarily represent those of their affiliated organizations, or those of the publisher, the editors and the reviewers. Any product that may be evaluated in this article, or claim that may be made by its manufacturer, is not guaranteed or endorsed by the publisher.

## Footnotes

1 JASP Team (2021). JASP (Version 0.16)[Computer software].

2 R Core Team (2021). R: A Language and Environment for Statistical Computing. Vienna: R Foundation for Statistical Computing. Available online at: https://www.R-project.org/.Version4.1.1packageRcmdrPLUGIN.survival.
